# Phenotypic and Genotypic Profiles of Extended-Spectrum Beta-Lactamase-Producing Multidrug-Resistant *Klebsiella pneumoniae* in Northeastern Thailand

**DOI:** 10.3390/antibiotics13100917

**Published:** 2024-09-25

**Authors:** Sumontha Chaisaeng, Nattamol Phetburom, Pachara Kasemsiri, Nuntiput Putthanachote, Naowarut Wangnadee, Parichart Boueroy, Anusak Kerdsin, Peechanika Chopjitt

**Affiliations:** 1 Faculty of Public Health, Kasetsart University Chalermphrakiat Sakon Nakhon Province Campus, Sakon Nakhon 47000, Thailand; sumontha.chai@ku.th (S.C.); nattamol.phet@ku.th (N.P.); parichart.bou@ku.th (P.B.); anusak.ke@ku.th (A.K.); 2 Clinical Microbiology Laboratory, Sakon Nakhon Hospital, Sakon Nakhon 47000, Thailand; nuipachara20@gmail.com; 3 Clinical Microbiology Laboratory, Roi-Et Hospital, Roi-Et 45000, Thailand; nuntiput101@gmail.com (N.P.); chefvy_123@hotmail.com (N.W.)

**Keywords:** *Enterobacterales*, ERIC-PCR, extended-spectrum β-lactamase, *Klebsiella pneumoniae*, multidrug resistance, third-generation cephalosporin resistance

## Abstract

The global emergence of extended-spectrum beta-lactamase (ESBL)-producing *Klebsiella pneumoniae* presents a significant public health threat and complicates antibiotic treatment for infections. This study aimed to determine the prevalence of ESBL-producing *K. pneumoniae* in a clinical setting, analyze their antimicrobial susceptibility profiles, and characterize both phenotypic and genetic determinants. A total of 507 non-duplicate clinical isolates of *Enterobacterales* were collected between 2019 and 2020, and third-generation cephalosporin resistance was screened by disk diffusion. Identification of *K. pneumoniae* was confirmed using biochemical tests and PCR with species-specific primers. Antimicrobial susceptibility testing was conducted using disk diffusion, and phenotypic ESBL production was confirmed using the combined disk method. Multiplex PCR detected ESBL genes (*bla*_TEM_, *bla*_SHV_, and *bla*_CTX-M_) and identified *bla*_CTX-M_ groups. The genetic relatedness of ESBL-producing strains was assessed using the ERIC-PCR approach. Fitty-four isolates were confirmed as ESBL producers, all classified as multidrug-resistant (MDR). All ESBL-producing *K. pneumoniae* isolates exhibited resistance to ampicillin and cefotaxime, with high resistance rates for ciprofloxacin (98.2%), azithromycin (94.4%), piperacillin–tazobactam (88.9%), and trimethoprim (83.3%). Genotypic analysis revealed *bla*_CTX-M_ was present in 94.4% of isolates, *bla*_SHV_ in 87%, and *bla*_TEM_ in 55.5%. The *bla*_CTX-M-1_ group was the most prevalent, accounting for 96.1% of isolates. Co-harboring of *bla*_CTX-M_, *bla*_SHV_, and *bla*_TEM_ occurred in 42.6% of isolates, with co-carrying of *bla*_CTX-M_, and *bla*_SHV_ was observed in 23/54 isolates. The ERIC-PCR analysis revealed 15 distinct types, indicating high genetic diversity. These findings highlight the urgent need for ongoing monitoring to control the spread of ESBL among *K. pneumoniae* and emphasize the importance of early detection and appropriate antibiotic selection for effectively treating infection caused by these pathogens.

## 1. Introduction

The global emergence of multidrug-resistant (MDR) bacteria poses a significant threat to public health, particularly in low-and middle-income countries (LMICs). Decades of inappropriate antibiotic use in human and animal health have exerted selective pressure, compromising the efficacy of beta-lactam antibiotics. In LMICs, which are a resource-limited setting, an inadequate antimicrobial stewardship program and unrestricted access to antibiotics have exacerbated this issue [1]. The indiscriminate use of third-generation cephalosporins has precipitated the rise of antimicrobial-resistant (AMR) organisms, notably resistant *E. coli* and *K. pneumoniae*, in both community and nosocomial settings. Consequently, AMR-associated hospital-acquired infections in LMICs’ ICUs exceed prevalence rates observed in high-income countries, complicating treatment strategies and threatening global health initiatives [2].

*Klebsiella pneumoniae* is a significant opportunistic pathogen in healthcare settings [3,4]. It is responsible for a wide range of infectious diseases, including pneumonia, bacteremia, sepsis, burn and wound infections, and urinary tract infections (UTIs) [5]. *K. pneumoniae* is primarily associated with hospital-acquired infections (HAIs), particularly among immunocompromised patients and those with underlying conditions such as diabetes, chronic obstructive pulmonary disease (COPD), or a history of antibiotic use [6]. The pathogen’s high prevalence and the ability of many strains to exhibit resistance to commonly used antibiotics, including carbapenems, have contributed to a notable increase in mortality and morbidity over the years [6]. *K. pneumoniae*’s capacity to acquire resistance genes across various antibiotic classes, such as the production of carbapenemases like NDM-1 and OXA-48, further enhances its pathogenic potential and leads to severe disease manifestations [7]. Consequently, the World Health Organization (WHO) has classified antibiotic-resistant *K. pneumoniae* as a Priority 1 (critical group) pathogen, indicating the urgent need for research and development of new antibiotics to combat this significant threat to global health [8].

*K. pneumoniae* exhibits resistance to beta-lactam antibiotics primarily through the production of beta-lactamases, particularly extended-spectrum beta-lactamases (ESBLs). These enzymes can hydrolyze first-, second-, and third-generation cephalosporins, as well as aztreonam, although they are inhibited by clavulanic acid. The coexistence of various modifying enzymes on the same plasmid can confer resistance to additional antibiotic classes, including fluoroquinolones, aminoglycosides, tetracyclines, and trimethoprim-sulfamethoxazole. ESBL-encoding genes are categorized into several families, with *bla*_TEM_ variants, *bla*_SHV_ variants, and *bla*_CTX-M_ being the most prevalent [9]. Among these, CTX-M enzymes, classified as class A ESBLs, are the most widely distributed worldwide, including in developing countries. These ESBL genes are typically located on plasmids but can occasionally be found on chromosomes. More than 50 allelic variants of CTX-M have been identified, which are grouped into six clusters: CTX-M-1, CTX-M-2, CTX-M-8, CTX-M-9, CTX-M-25, and KLUC based on a ≥10% variance in amino acid sequence identity, with several minor variants within the groups. CTX-M-type ESBLs are predominantly detected in plasmid incompatibility groups, although chromosomal integration has also been reported [10]. In humans, CTX-M-15 (CTX-M-1 group) and CTX-M-14 (CTX-M-9 group) are more prevalent, whereas CTX-M-1 (CTX-M-1 group) is more predominant in animals. Other CTX-M groups have been reported in specific regions, such as the CTX-M-2 and CTX-M-8 groups in South America and the CTX-M-2 group in Japan [11]. The most identified CTX-M groups in Gram-negative pathogens are CTX-M-1, CTX-M-2, CTX-M-8, and CTX-M-9 [12]. 

In Thailand, the prevalence of ESBL-producing *K. pneumoniae* ranges from 12.7% to 30.2% [13,14,15]. The *bla*_CTX-M_ is believed to be the dominant type in the Asia–Pacific region, contributing to dissemination and outbreak in several countries. Therefore, ongoing surveillance of ESBL-producing *K. pneumoniae* is crucial to monitor and develop effective countermeasures against potential nosocomial infections. This study aimed to characterize ESBL-producing *K. pneumoniae* isolates collected between 2019 and 2020 from two tertiary hospitals in northeastern Thailand. The objectives included determining the prevalence of ESBL-producing *K. pneumoniae*, assessing their antimicrobial susceptibility profiles, and characterizing their phenotypic and genetic features.

## 2. Results

### 2.1. Characteristics of the Recovered K. pneumoniae Isolates

Among 507 non-duplicate Enterobacterales clinical isolates, *K. pneumoniae* was identified in 70 isolates (13.8%) recovered from various clinical samples, including feces (*n* = 14), urine (*n* = 16), blood (*n* = 5), pus (*n* = 7), and sputum (*n* = 28). Phenotypically, *K. pneumoniae* is characterized as a Gram-negative, rod-shaped, non-motile bacterium that grows on MacConkey agar, producing mucoid colonies that are pink to red due to lactose fermentation. Additional phenotypic characteristics include indole positivity, urease negativity, citrate negativity, gas production, hydrogen sulfide negativity, and lysine decarboxylase positivity. 

### 2.2. Phenotypic Determination for ESBL-Producing K. pneumoniae Isolates 

**Figure 1:**
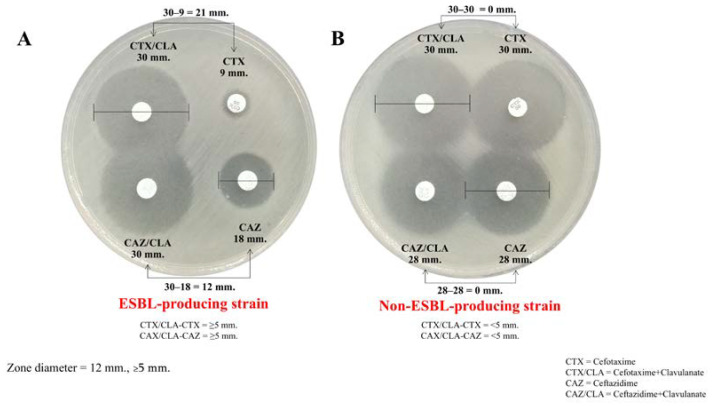
The combination disk method for ESBL detection. (**A**) Zone diameter: ≥5 mm difference between the cefotaxime (CTX) and cefotaxime/clavulanic acid (CTX/CLA) disks and the ceftazidime (CAZ) and ceftazidime/clavulanic acid (CAZ/CLA) disks, which suggests an ESBL-producing strain (top). (**B**) Zone diameter: <5 mm difference between the CTX and CTX/CLA disks and the CAZ and CAZ/CLA disks, which does not suggest an ESBL-producing strain.

All 70 *K. pneumoniae* isolates exhibited resistance, as determined by disk diffusion assays using cefotaxime (30 μg) and ceftazidime (30 μg). Among these, all *K. pneumoniae* isolates were resistant to cefotaxime, while 56 isolates (80%, 56/70) demonstrated resistance to both cefotaxime and ceftazidime. Further analysis using the combination disk method in Figure 1 revealed that 54 out of the 70 isolates (77.1%) produced ESBLs. The ESBL-producing *K. pneumoniae* isolates were recovered from various clinical specimens, including sputum (37.0%, *n* = 20/54), feces (25.9%, *n* = 14/54), urine (16.7%, *n* = 13/54), pus (7.4%, *n* = 4/54), and blood (5.5%, *n* = 3/54). 

### 2.3. Antimicrobial Susceptibility Pattern of ESBL-Producing K. pneumoniae Isolates 

**Figure 2:**
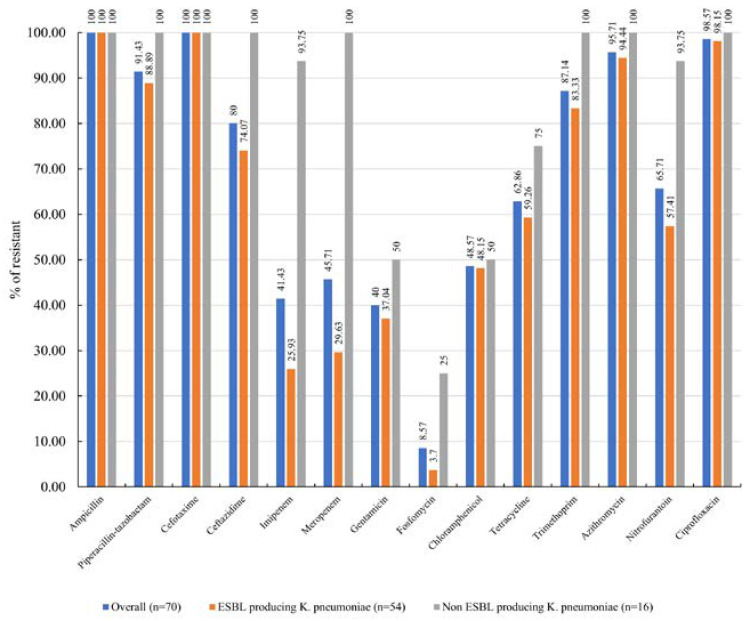
Antimicrobial resistance of ESBL-producing *K. pneumoniae* isolates.

The antimicrobial resistance profile of the ESBL-producing *K. pneumoniae* isolates is illustrated in Figure 2. All isolates exhibited complete resistance to ampicillin and cefotaxime, with relatively high resistance rates observed for ciprofloxacin (98.6%, 69/70), azithromycin (95.7%, 67/70), piperacillin-tazobactam (91.4%, 64/70), trimethoprim (87.1%, 61/70), ceftazidime (80%, 56/70), nitrofurantoin (65.7%, 46/70), and tetracycline (62.9%, 44/70). All ESBL-producing *K. pneumoniae* isolates were classified as multidrug-resistant (MDR), defined as resistance to at least one agent in three or more antimicrobial categories. Additionally, 14.8% (8/54) of the ESBL-producing *K. pneumoniae* isolates were identified as extensively drug-resistant (XDR), isolates resistant to at least one drug in all but two or fewer antimicrobial categories. The antimicrobial resistance patterns are presented in Table 1. 

### 2.4. Molecular Characterization of β-Lactamase Genes

**Figure 3:**
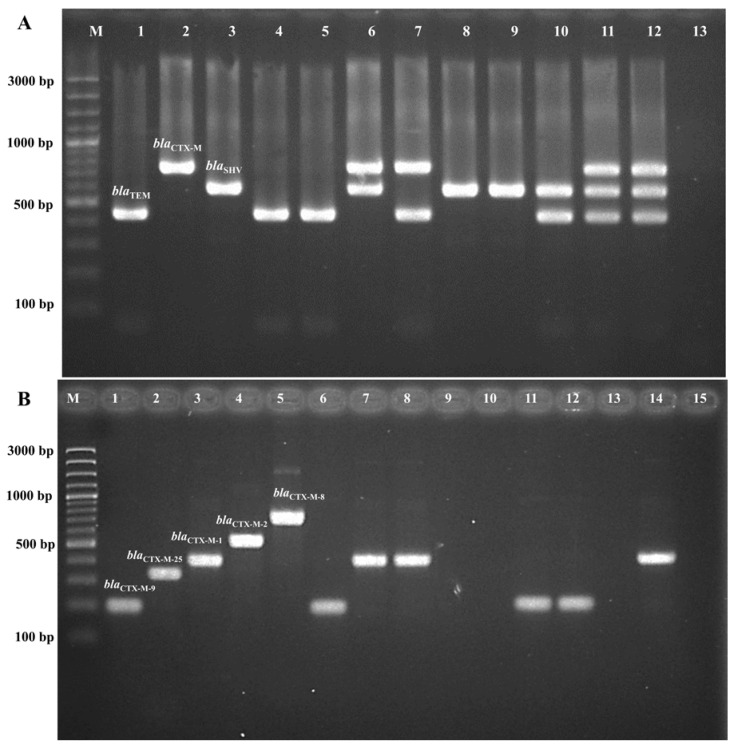
Multiplex PCR for ESBL gene detection. (**A**). Multiplex PCR for *bla*_TEM_, *bla*_SHV_, and *bla*_CTX-M_. Lane M is a 100 bp molecular weight marker, lane 1 is a *bla*_TEM_ positive control, lane 2 is a *bla*_SHV_ positive control, lane 3 is a *bla*_CTX-M_ positive control, lanes 4–12 are samples, and lane 13 is a negative control. (**B**). Multiplex PCR for *bla*_CTX-M_ groups, including *bla*_CTX-M-1_, *bla*_CTX-M-2_, *bla*_CTX-M-8_, *bla*_CTX-M-9_, and *bla*_CTX-M-25_. Lane M is a 100 bp molecular weight marker, lane 1 is a *bla*_CTX-M-1_ positive control, lane 2 is a *bla*_CTX-M-2_ positive control, lane 3 is a *bla*_CTX-M-8_ positive control, lane 4 is a *bla*_CTX-M-9_ positive control, lane 5 is a *bla*_CTX-M-25_ positive control, lanes 6–14 are samples, and lane 15 is a negative control.

The presence of beta-lactamase genes in all 54 ESBL-producing *K. pneumoniae* strains was determined using multiplex PCR, as shown in Figure 3. The predominant genes identified in our study were *bla*_CTX-M_ (94.4%, 51/54), followed by *bla*_SHV_ (87.0%, 47/54) and *bla*_TEM_ (55.5%, 30/54). The most prevalent combination patterns were *bla*_TEM_ + *bla*_CTX-M-1_ + *bla*_SHV_ and *bla*_CTX-M_ + *bla*_SHV_ (42.6%, 23/54). Among the 51 ESBL-producing *K. pneumoniae* isolates carrying *bla*_CTX-M_, the majority belonged to the *bla*_CTX-M-1_ group (96.0%, 49/54 isolates), while the *bla*_CTX-M-9_ group was found in only one isolate. One isolate carried an unidentified group of *bla*_CTX-M_ (Table 2).

### 2.5. ERIC-PCR Analysis

**Figure 4:**
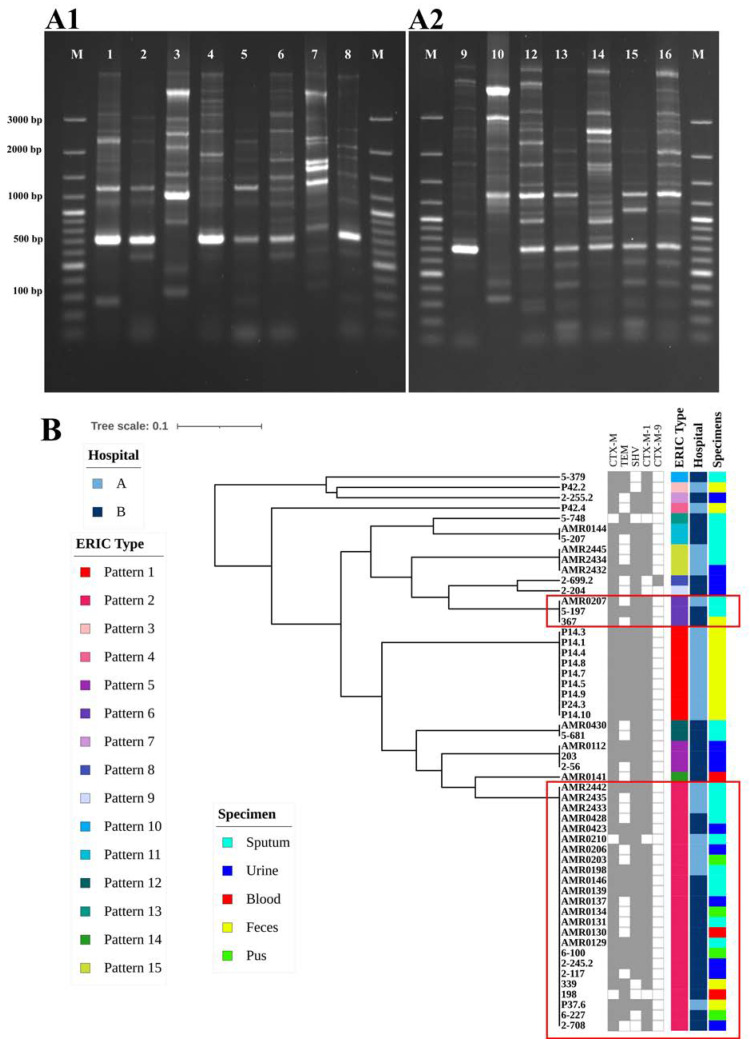
(**A**) (**A1**,**A2**). Gel electrophoresis analysis of ERIC-PCR profiles of ESBL-producing *K. pneumoniae* isolated in our study. (**B**) Dendrogram generated from ERIC-PCR fingerprinting for ESBL-producing *K. pneumoniae* isolated in our study. Fifty-four isolates were designated to 15 patterns.

The genetic relatedness among ESBL-producing *K. pneumoniae* isolates was analyzed using ERIC-PCR; the analysis identified 15 different ERIC types, including seven common types and eight unique types (Figure 4(A1,A2)). A total of 15 different ERIC profiles (E-types) were observed, designated as P1 to P15. Out of 54 isolates, 24 belonged to P2, 9 were P1, and 3 each were P5, P6, and P15. Additionally, two isolates were categorized as P11 and P12, while eight isolates exhibited unique patterns. At a 95% similarity level, these isolates were classified into two clusters: A and B. The predominant cluster B contained 51 isolates (94%), while cluster A contained 3 isolates (5.5%) (Figure 4B). Notably, the strains of AMR0207 from hospital A and 5-197 and 367 from hospital B displayed the same ERIC-PCR pattern (P6). Furthermore, strains of AMR2442, AMR2435, AMR2433, AMR0210, AMR0206, AMR0203, AMR0198, and P37.6 from hospital A and AMR0428, AMR0423, AMR0146, AMR0139, AMR0137, AMR0134, AMR0131, AMR0130, AMR0129, 6-100, 2-245.2, 2-117, 339, 198, P37.6, 6-227, and 2-708 from hospital B revealed the same pattern of ERIC-PCR (P2). These results suggest the possibility of clonal distribution between hospital A and hospital B.

## 3. Discussion

The widespread emergence of ESBLs in pathogenic bacteria presents a significant public health concern. These enzymes confer resistance to a broad spectrum of beta-lactam antibiotics, compromising their efficacy and creating therapeutic challenges. This situation necessitates the development of reliable detection methods in clinical microbiology laboratories to guide appropriate antimicrobial therapy and infection control measures. Notably, *K. pneumoniae*, a major nosocomial pathogen, exhibits a high prevalence of ESBL production, further underscoring the need for effective detection strategies. 

The present study revealed a prevalence of 10.7% (54/507) for ESBL-producing *K. pneumoniae* among *Enterobacterales* clinical isolates. This finding is similar to the 10.2% prevalence reported by Siriphap et al. in northern Thailand [13] and the 7.5% prevalence found by Ko et al. in the Asia–Pacific region, including Thailand [16]. However, it is notably lower than the 27.4% prevalence described by Sawatwong et al. in a different region of Thailand [15], suggesting potential variability within the country. Additionally, the prevalence of ESBL-producing Gram-negative bacteria or *Enterobacterales* was 24% in Egypt [17], 27.6% in Nigeria [18], 29.6% in Cameroon [19],15.3% in Ethiopia [20], 14.3% in India [21], and 16.8% in Nepal [22]. Upon recalculating the prevalence rates based on data from those studies, the prevalence of ESBL-producing *K. pneumoniae* is comparable to our current study finding, with rates of 11.4% (12/105) in Egypt [17], 12% (26/217) in Nigeria [18], 13.5% (19/141) in Ethiopia [20], 14.3% (72/504) in India [21], and 15% (322/2153) in Nepal [22]. Conversely, our findings indicate a higher prevalence compared to studies conducted in Cameroon, at 5.3% (8/152) [19], and Japan, at 4.8% (144/2987) [23]. These variations in prevalence across different studies and geographical regions may be attributed to multiple factors, including differences in study methodologies, such as sample size, sampling technique, participant demographics, antibiotic usage patterns, and regional infection control practices. Further investigation into these contributing factors could provide valuable insights into the global epidemiology of ESBL-producing *K. pneumoniae.*

Among the fifty-four ESBL-producing *K. pneumoniae* isolates, sputum samples (37.0%) constituted the predominant source, followed by feces, urine, pus, and blood. This result is comparable to studies conducted in northern Thailand (44.2%) [13]. However, several studies have identified urine as the major reservoir of ESBL producers, including investigations conducted in southern Thailand [24], northern Portugal [25], Nigeria [26], Uganda [27], and Ghana [28]. Additionally, some studies have reported blood [29] and feces [30] as the main sources of ESBL-producing *K. pneumoniae*. These variations likely reflect differences in sampling techniques, specimen collection, patient demographics, and geographic distribution. 

The high resistance profile observed for penicillin and cephalosporin in our studies is consistent with findings from other studies [24,31,32,33,34,35]. This resistance can be attributed to the excessive use of amoxicillin and cefotaxime, ceftriaxone, and ceftazidime in hospitals, where hospitalization itself has been identified as a risk factor for infection with ESBL-producing *Enterobacterales*. Plasmids carrying genes that encode ESBLs can be easily transmitted horizontally between different bacteria in the hospital environment. Our study revealed an association between ESBL production and resistance to multiple antibiotic classes. 

According to our finding, the overall prevalence of MDR among ESBL-producing *K. pneumoniae* isolates was 100%. This finding aligns with studies conducted in Ethiopia, which reported an MDR rate of 100% and 93.6% [36,37] and a 96.4% MDR rate in Ghana [34]. Moreover, our study shows that 14.8% of ESBL-producing *K. pneumoniae* isolates were XDR, which is lower than the 41% reported by Romyasamit C et al. in Southern Thailand [24].

Among the ESBL-producing *K. pneumoniae* isolates, the *bla*_CTX-M_ gene was the most frequently detected, which is consistent with other studies, such as 83.1% in Nigeria [38] and 61.5% in Japan [39]. In our study, the prevalence of the *bla*_CTX-M_ gene was 94.4% (Table 2). This result aligns with data obtained from the central part of Thailand, where the prevalence of *bla*_CTX-M_ among clinical ESBL-*K. pneumoniae* was 99.2% (126/127) [14], and from Nigeria at 93.3% (98/105) [38], Japan at 97% (131/135) [39], Malaysia at 93.5% (87/93) [31], Brazil at 67.8% (120/177) [40], and China at 90.8% (167/184) [41]. The importance of genetic studies of ESBL genes lies in their capacity to spread horizontally to other bacterial species. This horizontal transfer leads to widespread ESBL activity among different pathogens in hospitals. The ongoing surveillance of genotypes in ESBL-producing *K. pneumoniae* is essential to monitor the emergence of new resistant clones in various regions. In our study, the *bla*_CTX-M-1_ group was the primary ESBL genotype identified among ESBL-producing *K. pneumoniae*. Our results are consistent with the predominance of the *bla*_CTX-M-1_ group report in Japan [23,39], China [41], the United States [42], Canada [29], and Ghana [43]. 

Additionally, we present evidence suggesting the potential of the same ESBL-producing *K. pneumoniae* clones (P2 and P6) between hospitals. This may indicate that these clones are commonly distributed and disseminated among human and non-human subjects in the community [41] or through a patient referral system between the hospitals [30,44]. Our study identified 15 distinct ERIC types, highlighting the genetic diversity of *K. pneumoniae* isolated from two hospitals. The prevalence of type P2 in both hospitals suggests that this clone may represent a major strain disseminating across multiple healthcare facilities. However, it is important to note that ERIC-PCR has limitations for *K. pneumoniae* typing. This method exhibits low discriminatory power. Therefore, complementary techniques such as pulsed-field gel electrophoresis (PFGE) or whole-genome sequencing could offer further insights into the genetic relationship among these isolates. However, we could tentatively cluster our isolates in the current study to guide clonal relationships. 

The limitations of our study are noteworthy. First, this research was conducted in only two tertiary hospitals in northeastern Thailand, which may not be representative of the prevalence of ESBL-producing *Klebsiella pneumoniae* isolates in the region. Sample size, types of samples collected, and the restricted time frame further constrain the generalizability of our findings. It would be beneficial to extend this study to encompass a broader geographic area to obtain more relevant results. In addition, we identified 70 *K. pneumoniae* strains among 507 *Enterobacterales* isolates, and 54 out of 70 were classified as ESBL producers. The low prevalence of *K. pneumoniae* in our study may be influenced by the types and quantities of samples collected. Furthermore, the CTX-M variants were not sequenced in this study, which restricts our understanding of the genetic diversity of these CTX-M. These limitations highlight the necessity for large-scale, multicenter prospective studies employing standardized methodologies and comprehensive molecular analyses to better elucidate the epidemiology, resistance mechanisms, and clinical implications of ESBL-producing *K. pneumoniae*.

## 4. Materials and Methods

### 4.1. Bacterial Isolation and Identification

Among the 507 non-duplicate clinical isolates of *Enterobacterales* obtained from various clinical specimens, including urine (*n* = 229), feces (*n* = 142), blood (39), pus (*n* = 52), and sputum (*n* = 45), between January 2019 and December 2020, the isolates were collected from two tertiary care hospitals located in a rural region of northeastern Thailand. The identification of *K. pneumoniae* was performed using a conventional method. The isolates were cultured on MacConkey agar plates (HIMEDIA, Mumbai, India) and incubated at 37 °C for 24 h before undergoing Gram staining. Presumptive identification of *K. pneumoniae* was conducted using biochemical tests, including catalase, oxidase, IMViC (indole, methyl red, Voges–Proskauer, citrate utilization), triple sugar iron, urease, motility, and oxidative fermentation tests [45]. Subsequently, species confirmation was performed by a polymerase chain reaction (PCR) assay using specific primers [46]. The primer sequence and conditions are listed in Table 3. The reaction mixture contained PCRBIO HS Taq DNA Polymerase & Mixes (London, UK), 0.3 µM of each primer, sterile deionized water, and 50 ng of bacterial DNA template. PCR was performed using a Thermal Cycler (Bio-Rad, Hercules, CA, USA) with the following program: initial denaturation at 95 °C for 3 min, followed by 30 cycles of denaturation at 95 °C for 30 s, annealing at 60 °C for 30 s and extension at 72 °C for 45 s, and a final extension of amplification at 72 °C for 5 min. The PCR products were analyzed using gel electrophoresis on 1.5% (*w*/*v*) agarose gel in 0.5× TBE buffer at a constant voltage of 100 V for 30 min (Mupid exU system; Takara; Tokyo, Japan). The gels were stained with ethidium bromide and visualized under ultraviolet light (GeneGenius Bioimaging System; SynGene; Cambridge, UK). The sizes of the PCR products were determined by comparison with molecular size standards (GeneRuler™ 100 bp Plus DNA ladder; Thermo Fisher Scientific; Vilnius, Lithuania). *K. pneumoniae* was cultured and inoculated into 20% glycerol–tryptic soy broth and stored in a −80 °C freezer. 

### 4.2. Phenotypic Detection of ESBL-Producing K. pneumoniae

*K. pneumoniae* strains were initially screened for third-generation cephalosporin resistance using the disk diffusion assay with ceftazidime (30 μg) and cefotaxime (30 μg). The cephalosporin-resistant isolates were non-susceptible (intermediate or resistant) to cefotaxime or ceftazidime, or both. The ESBL production was determined using the combination disk method according to the guidelines of the Clinical and Laboratory Standards Institute [50]. Briefly, bacterial cultures adjusted to a 0.5 McFarland standard were inoculated onto Mueller–Hinton agar plates (Merck, Frankfurter, Germany). Ceftazidime (30 µg), ceftazidime/clavulanate (30/10 µg), cefotaxime (30 µg), and cefotaxime/clavulanate (30/10 µg) disks (MASTDISCS^®^AST, Mereyside, UK) were placed on the inoculated plates, which were incubated at 37 °C for 18 h. The isolates that exhibited ≥5 mm inhibition zones with combination disks compared to individual antibiotics (without clavulanate) were designated as ESBL producers.

### 4.3. Antimicrobial Susceptibility Testing of K. pneumoniae 

The antimicrobial susceptibility of the isolates was assessed following the CLSI 2023 guidelines. The following antibiotic disks (MASTDISCS^®^AST, Mereyside, UK) were used: piperacillin–tazobactam (PTZ 110 µg), ampicillin (AP 10 µg), ceftazidime (CAZ 30 µg), cefotaxime (CTX 30 µg), ciprofloxacin (CIP 5 µg), gentamicin (GM 10 µg), imipenem (IMP; 10 µg), meropenem (MEM; 10 µg), tetracycline (T 30 µg), chloramphenicol (C 30 µg), trimethoprim (TM 15 µg), and nitrofurantoin (NI 300 µg). The plates were incubated at 37 °C for 18–24 h. After incubation, the zones of inhibition were measured and interpreted as susceptible, intermediate, or resistant based on CLSI recommendations [50]. Isolates resistant to two or more classes of antimicrobial agents were classified as multidrug-resistant (MDR) according to the guidelines described by Magiorakos et al. [51].

### 4.4. DNA Extraction and Quantification 

Genomic DNA from ESBL-producing *K. pneumoniae* strains was extracted using a ZymoBIOMICs^TM^ DNA Miniprep Kit (Zymo Research; Irvine, CA, USA) following the manufacturer’s instructions. The extracted DNA was stored at −20 °C until further analysis. To quantify the DNA concentration and assess its purity, absorbance readings were taken at 260 and 280 nm using a NanoDrop^TM^ 2000 Spectrophotometer (Thermo Fisher Scientific; Waltham, MA, USA).

### 4.5. Molecular Detection of Beta-Lactamase Genes by PCR 

The presence of *bla*_TEM_, *bla*_SHV_, *bla*_CTX-M_, and the *bla*_CTX-M_ subgroup (*bla*_CTX-M_ groups 1, 2, 8, 9, and 25) in ESBL-producing isolates was determined using multiplex PCR, following previously described protocols [47,48]. All primers were synthesized by Integrated DNA Technologies (Singapore). The target genes, primers sequence, cycle conditions, and expected amplicon size are described in Table 3. PCR was conducted using a T100 thermal cycler (BioRad, Hercules, CA, USA) with a total volume of 25 µL, which included PCRBIO HS Taq DNA Polymerase & Mixes (London, UK), 0.3 µM of each primer, sterile deionized water, and 50 ng of DNA template. The PCR amplification program consisted of an initial denaturation at 95 °C for 3 min, followed by 30 cycles of denaturation at 95 °C for 30 s, annealing at 55 °C for 30 s, extension at 72 °C for 1 min, and a final amplification at 72 °C for 7 min. For the *bla*_CTX-M_ subgroup, the PCR conditions were modified to include an initial denaturation at 94 °C for 5 min, followed by 30 cycles of denaturation at 94 °C for 25 s, annealing at 52 °C for 40 s, extension at 72 °C for 50 s, and a final amplification at 72 °C for 6 min. 

The PCR products from each reaction were subjected to electrophoresis on 1.5% agarose gel alongside a DNA marker (GeneRuler™ 100 bp Plus DNA ladder; Thermo Fisher Scientific; Vilnius, Lithuania). The gels were visualized and photographed under UV transillumination (SYNGENE, Cambridge, UK).

### 4.6. Quality Control

For the standardization of the drug susceptibility test, *K. pneumoniae* (ATCC 2524) and *E. coli* (ATCC 25922) were used as the control strains. For the PCR controls, sterile water served as the negative control, while known positive DNA and negative controls from the previous extraction were processed to ensure the accuracy of the PCR procedure.

### 4.7. Enterobacterial Repetitive Intergenic Consensus–Polymerase Chain Reaction (ERIC-PCR) 

Genetic relatedness among the fifty-four ESBL-producing *K. pneumoniae* strains was assessed using ERIC-PCR with ERIC-1 and ERIC-2 primers [49]. The sequence primers are shown in Table 3. The reaction mixture contained PCRBIO HS Taq DNA Polymerase & Mixes (London, UK), 0.5 µM of each primer, sterile deionized water, and 50 ng of bacterial DNA template. The cycling conditions were as follows: an initial denaturation at 95 °C for 3 min, followed by 40 cycles of denaturation at 92 °C for 30 s, annealing at 52 °C for 1 min, extension at 72 °C for 8 min, and a final extension at 72 °C for 16 min. The ERIC-PCR fragments were visualized by 1% agarose gel electrophoresis stained with GelRed (Biotium, Inc., Hayward, CA, USA). 

The pattern of DNA fingerprints was visually compared, and patterns differing by at least one amplification band were classified as different. For constructing a computerized dendrogram, the presence and absence of bands were recorded as 1 and 0, respectively. ERIC profiles were compared using the Dice similarity matrix coefficient and clustered by the neighbor-joining method to prepare the phylogenetic tree. Isolates exhibiting two or more different bands in the ERIC banding pattern were considered different ERIC types. The dendrogram was constructed using the FreeTree program, employing an unweighted paired group method with arithmetic mean (UPGMA) according to published guidelines by [52]. The UPGMA tree was visualized by iToLv5 [53]. 

## 5. Conclusions

The findings of this study confirm a high prevalence rate of 94.4% for ESBL-producing *K. pneumoniae*, with a significant proportion of isolates exhibiting MDR. The highest levels of resistance were observed against ampicillin and cefotaxime, with the *bla*_CTX-M-1_ group identified as the most prevalent ESBL gene. These results align with broader concerns regarding antibiotic-resistant ESKAPE pathogens, which are associated with increased mortality risk and economic costs, particularly in developing countries. Furthermore, there is a pressing need for broader studies and ongoing surveillance to monitor resistance patterns beyond the geographic and temporal limitations of this research. Routine screening for ESBL-producing pathogens is crucial for early detection and appropriate antibiotic selection. On a larger scale, developing rapid diagnostics, new antibiotics, and vaccines, along with creating an international platform for real-time surveillance of antimicrobial resistance, are vital steps in containing this global threat and ensuring high-quality, effective therapeutic options. 

## Figures and Tables

**Table 1:** Antimicrobial resistance patterns of ESBL-producing *K. pneumoniae* isolates (*n* = 54).

Pattern Number	Antimicrobial Resistance Patterns	Number of Isolates	Type of Resistance	
1	AP+CTX+CAZ+C+TM+ATH+CIP	1	MDR	
2	AP+CTX+CAZ+CIP	1	MDR	
3	AP+CTX+CAZ+IMP+MEM+T+ATH+NI+CIP	1	MDR	
4	AP+CTX+CAZ+T+TM+ATH+NI+CIP	1	MDR	
5	AP+CTX+FOT+C+TM+ATH+NI+CIP	1	MDR	
6	AP+CTX+T+TM+ATH+NI+CIP	1	MDR	
7	AP+PTZ+CTX+CAZ+C+T+TM+ATH+NI+CIP	1	MDR	
8	AP+PTZ+CTX+CAZ+GM+C+CIP	1	MDR	
9	AP+PTZ+CTX+CAZ+GM+C+T+TM+ATH+CIP	1	MDR	
10	AP+PTZ+CTX+CAZ+GM+C+T+TM+ATH+NI+CIP	1		XDR
11	AP+PTZ+CTX+CAZ+GM+C+TM+ATH+CIP	7	MDR	
12	AP+PTZ+CTX+CAZ+GM+C+TM+ATH+NI+CIP	1	MDR	
13	AP+PTZ+CTX+CAZ+GM+T+ATH+NI+CIP	1	MDR	
14	AP+PTZ+CTX+CAZ+GM+TM+ATH+CIP	1	MDR	
15	AP+PTZ+CTX+CAZ+IMP+MEM+ATH+NI+CIP	1	MDR	
16	AP+PTZ+CTX+CAZ+IMP+MEM+C+ATH+NI+CIP	2	MDR	
17	AP+PTZ+CTX+CAZ+IMP+MEM+C+T+ATH+NI+CIP	1	MDR	
18	AP+PTZ+CTX+CAZ+IMP+MEM+C+T+TM+ATH+NI+CIP	2		XDR
19	AP+PTZ+CTX+CAZ+IMP+MEM+C+TM+ATH+NI+CIP	1	MDR	
20	AP+PTZ+CTX+CAZ+IMP+MEM+GM+C+T+TM+ATH+NI+CIP	1		XDR
21	AP+PTZ+CTX+CAZ+IMP+MEM+T+TM+ATH+CIP	3	MDR	
22	AP+PTZ+CTX+CAZ+IMP+MEM+T+TM+ATH+NI+CIP	1	MDR	
23	AP+PTZ+CTX+CAZ+T	1	MDR	
24	AP+PTZ+CTX+CAZ+T+TM+ATH+CIP	4	MDR	
25	AP+PTZ+CTX+CAZ+T+TM+ATH+NI+CIP	3	MDR	
26	AP+PTZ+CTX+CAZ+TM+ATH+NI+CIP	1	MDR	
27	AP+PTZ+CTX+FOT+C+TM+CIP	1	MDR	
28	AP+PTZ+CTX+GM+C+T+TM+ATH+NI+CIP	4		XDR
29	AP+PTZ+CTX+GM+T+ATH+NI+CIP	1	MDR	
30	AP+PTZ+CTX+GM+TM+ATH+CIP	1	MDR	
31	AP+PTZ+CTX+IMP+MEM+TM+ATH+NI+CIP	1	MDR	
32	AP+PTZ+CTX+T+TM+ATH+CIP	1	MDR	
33	AP+PTZ+CTX+T+TM+ATH+NI+CIP	3	MDR	
34	AP+PTZ+CTX+TM+ATH+NI+CIP	1	MDR	
	Total	54	54 (100)	8 (14.81)

Abbreviations: ampicillin, AP; gentamicin, GM; piperacillin–tazobactam, PTZ; cefotaxime, CTX; ciprofloxacin, CIP; ceftazidime, CAZ; chloramphenicol, C; tetracycline, T; nitrofurantoin, NI; azithromycin, ATH; trimethoprim, TM; imipenem, IMP; meropenem, MEM; MDR, multidrug-resistant; and XDR, extensively drug-resistant.

**Table 2:** Distribution of β-lactamase genes in ESBL-producing *K. pneumoniae* isolates in clinical specimens.

ESBL Gene Pattern	ESBL-Producing *K. pneumoniae* *n* (%)
*bla*_TEM_	2 (3.7)
*bla*_CTX-M1_	1 (1.9)
*bla*_TEM_+*bla*_SHV_	1 (1.9)
*bla*_TEM_+*bla*_CTX-M_	4 (7.4)
*bla*_CTX-M_+*bla*_SHV_	23 (42.6)
*bla*_TEM_+*bla*_CTX-M1_+*bla*_SHV_	23 (42.6)
Total	54
Total *bla*_TEM_	30 (55.5)
Total *bla*_CTX-M_	51 (94.4)
Total *bla*_SHV_	47 (87.0)

**Table 3:** Primer sequences for PCR detection in this study.

Gene	Primer Sequence (5′-3-)	Product Size (bp)	Reference
*K. pneumoniae*	F: CGGATCCTGGTCATTAAGCTG	217	[46]
	R: ATTGCATCTTCAGCTGATACCTTT		
*bla*_TEM_	F: ACGCTCACCGGCTCCAGATTAT	445	[47]
	R: TCGCCGCATACACTATTCTCAGA		
*bla*_CTX-M_	F: ATGTGAGYACCAGTAARGTGAT	593	
	R: TGGGTRAARTARGTSACCAGAAT		
*bla*_SHV_	F: TGCTTTGTTATTCGGGCCAA	747	
	R: ATGCGTTATATTCGCTGTG		
*bla*_CTX-M-1_	F: AAAAATCACTGCGCCAGTTC	415	[48]
	R: AGCTTATTCATCGCCACGTT		
*bla*_CTX-M-2_	F: CGACGCTACCCCTGCTAT T	552	
	R: CCAGCGTCAGATTTTTCAGG		
*bla*_CTX-M-8_	F: TCGCGTTAAGCGGATGATGC	666	
	R: AACCCACGATGTGGGTAGC		
*bla*_CTX-M-9_	F: CAAAGAGAGTGCAACGGATG	205	
	R: ATTGGAAAGCGTTCATCACC		
*bla*_CTX-M-25_	F: GCACGATGACATTCGGG	327	
	R: AACCCACGATGTGGGTAGC		
ERIC	F: AAGTAAGTGACTGGGGTGAGCG		[49]
	R: ATGTAAGCTCCTGGGGATTCAA		

## Data Availability

Data are contained within the article.
